# Physical discomforts, feeling of the high work intensity and the related risk factors of the frontline medical staff during COVID-19 epidemic: an early-outbreak, national survey in China

**DOI:** 10.3389/fpubh.2023.1270366

**Published:** 2023-10-12

**Authors:** Liu Jia, Ming Ye, Hongliang Wang, Huaiquan Wang

**Affiliations:** Department of Intensive Care Medicine, The Second Affiliated Hospital of Harbin Medical University, Harbin, China

**Keywords:** COVID-19, medical staff, physical discomforts, work intensity, risk factors

## Abstract

**Background:**

Facing the unknown virus, COVID-19 medical staff kept wearing thick personal protective equipment during their work in the early stage of the outbreak. The survey was designed to investigate the physical discomforts, the feeling of the work intensity and the related risk factors of the frontline medical staff during COVID-19 epidemic in the early outbreak.

**Methods:**

An national survey was carried out in China from March 17th 2020 to March 20th 2020 by applying a standardized WeChat questionnaire survey. The doctors or nurses working in the wards for the confirmed COVID-19 patients on front-line were eligible to participate in the survey. Descriptive analysis and multivariate logistic regression analysis were used.

**Results:**

A total number of 515 COVID-19 medical staff, including 190 physicians and 325 nurses participated in this survey. 375 medical staff (72.8%) experienced physical discomforts at work, mostly consist of dyspnea (45.8%), pain (41.0%), chest distress (24.1%), dizziness (18.8%), and weakness (17.5%), while wearing thick isolation clothes at work. The mean onset time and peak time of these symptoms were 2.4 h and 3.5 h after working, respectively. 337 medical staff (65.4%) suffered from sleep disorders. 51 medical staff (10%) were highly worried about being infected by COVID-19 even during their work breaks. 246 medical staffs (47.8%) felt high work intensity and the independent influential factors were the effective daily sleep time and anxiety levels at break time (*p* = 0.04).

**Conclusion:**

The frontline medical staff during COVID-19 epidemic felt different physical discomforts when they wear thick isolation clothes at work in the early outbreak and they felt high work intensity. These precious data will help optimize the work management strategy to ensure the physical and mental health of medical staff in the face of similar outbreaks in future.

## 1. Introduction

Since the outbreak of 2019 novel coronavirus (COVID-19) in Wuhan, Hubei province, China in late December 2019 (1–5), COVID-19 cases are still being continuously confirmed all over the world (6). This disease was transmitting so fast that the health-care system had been facing a sudden crisis. Moreover, the mortality rate was considerable in critically ill patients, as high as 61·5% (7). It is no doubt a huge challenge for medical staff never met before.

**Figure 1:**
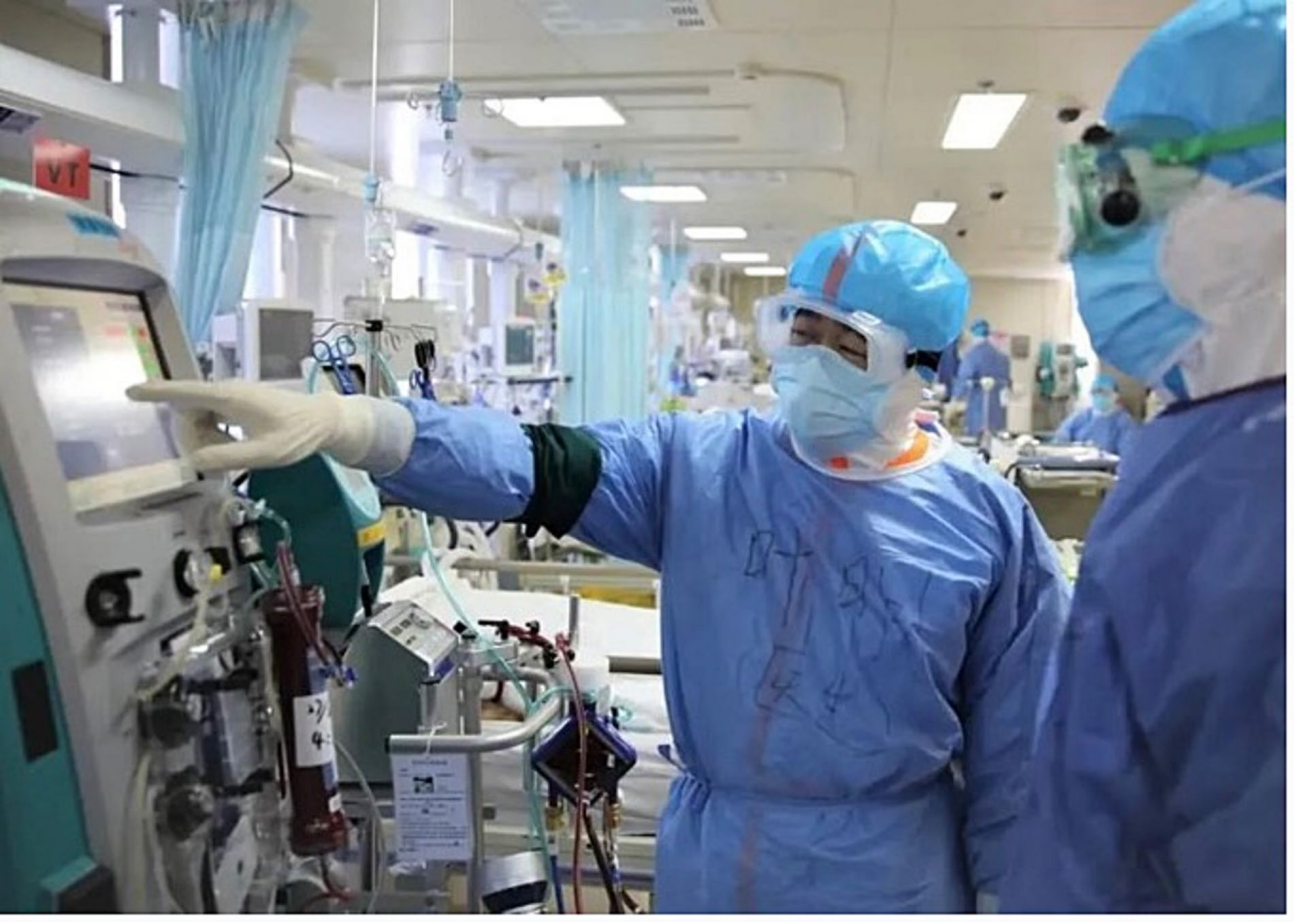
The COVID-19 medical staff wore thick personal protective equipment (PPE).

Reports showed that many health-care workers had been infected by COVID-19, and some of them had died (8–11). The mental stress of the health-care workers increased significantly when they cared for a large number of anxious COVID-19 patients with high-intensity work (12, 13). Lai (14) reported that health-care workers experiencing psychological burden, directly engaged in the diagnosis, treatment, and care for patients with COVID-19. Therefore, the medical staff for COVID-19 patients wore thick personal protective equipment (PPE) to protect themselves not being infected in the early outbreak in China, including three layers of medical hats, two layers of medical masks (N95 and surgical mask), eye protection (goggles or face screens), two layers of waterproof isolation clothing (a long fluid-impermeable gown and an operating coat), two layers of gloves, and two layers of shoe covers (Figure 1). This combination of PPE may cause increased work of breathing, reduced field of vision, muffled speech, difficulty hearing, and heat stress (15). Also, the medical staff who care for patients infected with COVID-19 are at a high risk of pressure injuries that caused by protective equipment in the prevention process (16). A growing concern regarding skin problems has been identified among healthcare workers during the COVID-19 pandemic (17–19), and the PPE-related skin injury can be serious (20). Daye (21) reported that skin problems were found to be 90.2%, the most common were dryness, itching, cracking, burning, flaking, peeling and lichenification. Severity of skin reaction was found to be significantly related to “hours per day of PPE use,” “consecutive days of PPE use,” and “female sex” (22). In the study by Proietti et al., prolonged use of PPE was a significant risk factor for developing skin related adverse events considering all the PPE considered (23). These occupational dermatoses caused by PPE in the ongoing COVID-19 pandemic are emerging occupational health challenges (24).

Therefore, the frontline medical staff faced great work stress and physical challenges during COVID-19 epidemic in the early outbreak. However, their physical discomforts and the feeling of the work intensity were not detailed described in previous studies. The survey was to comprehensively investigate their physical discomforts, the feeling of the work intensity and the related risk factors. When people face similar outbreaks in the future, these precious data may be learned from by the medical workers.

## 2. Methods

### 2.1. Study design and data collection

**Figure 2:**
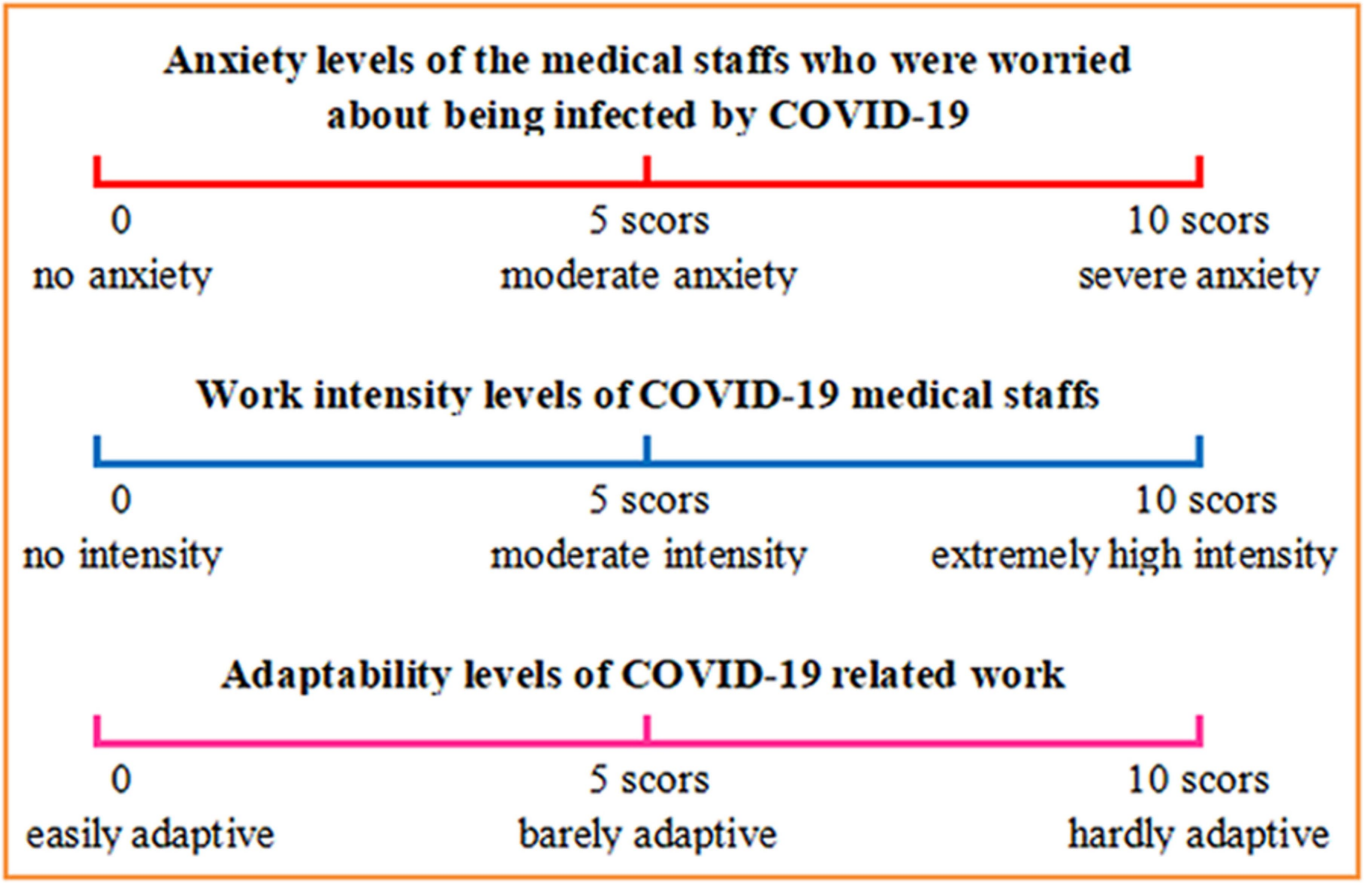
Visual Analogue Scale (VAS) used in the investigation.

An anonymous investigation was carried out in China from March 17th 2020 to March 20th 2020 by applying a standardized anonymous WeChat questionnaire and the details are provided in the Supplementary material. The medical staff directly taking care of the confirmed COVID-19 patients were eligible to participate in the survey. The questionnaire consists of three parts. The first part is to collect basic characteristics, including demographic information and general work history. The second part is to investigate the physical discomforts of the COVID-19 medical staff at work. Other work related information were also included, such as work location, personal protective status, work time. The third part of the questionnaire collects information about the feeling of the work intensity and other mental state. Visual Analogue Scale (VAS) was used to evaluate the feeling of the work intensity levels, the anxiety levels of being infected by COVID-19 both at work and break time, and the adaptability levels to the COVID-19 related work (Figure 2). The feeling of the work intensity levels were further categorized into two groups according to the VAS scores, low-moderate intensity (VAS score: zero-five) and high intensity (VAS score: six-ten). Sleep disorder and the psychological interventions during the COVID-19 work period were also investigated.

### 2.2. Statistical analysis

Statistical analyses were performed using SPSS 22.0 software (SPSS Inc., Chicago, IL, United States). Quantitative variables were reported as mean with standard deviation or median with interquartile spacing (IQR). Qualitative data were described as values or percentages. A *p* < 0.05 was considered statistically significant.

Potential influential factors for feeling of the work intensity were identified firstly by univariate logistic regression analysis. Those factors with p < 0.05 were further included in a stepwise multivariate logistic regression analysis. Results were reported as the odds ratio (*OR*) with 95% confidence interval (CI).

## 3. Results

### 3.1. Basic characteristics

A total number of 515 medical staff for COVID-19 [mean age, 34.5 (SD, 7.1) years; mean weight, 58.8 (SD, 18.0) kg; 190 (36.9%) physicians and 325 (63.1%) nurses], participated in this anonymous survey. As shown in Table 1, 389 medical staff (75.5%) came from Heilongjiang province, and 126 medical staff (24.5%) were from other provinces in China. 198 ICU medical staff accounted for 38.4% of all the participants in this survey. The rest of them were from respiratory department (63 medical staff, 12.2%), infectious disease department (15 medical staff, 2.9%), emergency department (11 medical staff, 2.1%), and other departments (228 medical staff, 44.3%). The medical staff mainly consisted of resident physicians and nurses (239 medical staff, 46.4%) and attending physicians and nurses (162 medical staff, 31.5%). Half of them had more than 10 years of work experience. The results showed that 39 medical staff (7.6%) had underlying physical diseases, such as hypertension or diabetes.

**Table 1:** Basic characteristics of 515 COVID-19 medical staffs.

	Median	IQR	Mean ± SD	Number	%*N*
Age, years	33	9	34.5 ± 7.1		
Gender					
Male				168	32.6
Female				347	67.4
Weight, kg	60	15	58.8 ± 18.0		
Hometown					
Heilongjiang providence				389	75.5
Other providences				126	24.5
Disciplines					
Intensive care unit				198	38.4
Respiratory department				63	12.2
Infectious disease department				15	2.9
Emergency department				11	2.1
Other departments				228	44.3
Type of staff					
Doctors				190	36.9
Nurses				325	63.1
Professional titles					
Resident physicians and nurses				239	46.4
Attending physicians and nurses				162	31.5
Associate chief physicians and nurses				74	14.4
Chief physicians and nurses				40	7.8
Working years					
<5 years				81	15.7
5–10 years				174	33.8
>10 years				260	50.5
Physical disease				39	7.6
Hypertension				6	1.2
Diabetes				3	0.6
Other problems				30	5.8

COVID-19 = 2019 novel coronavirus.

### 3.2. Physical discomforts and other work related information

Table 2 shows the work related information of the medical staff for COVID-19. All of them worn thick PPE at work (Figure 1). Most of them worked in Heilongjiang province (257 medical staff, 49.9%) or Hubei province (227 medical staff, 44.1%). None of the medical staff in this study was infected with COVID-19. Upon the time of the survey, these medical staff had continued working for COVID-19 patients for mean 26.3 [SD, 13.4] days. Nearly half of the medical staff (229 medical staff, 44.5%) had finished their rescue work for COVID-19 patients at the time of data collection.

**Table 2:** Work status of 515 COVID-19 medical staffs.

	Median	IQR	Mean ± SD	Number	%*N*
Work location					
Heilongjiang province				257	49.9
Hubei province				227	44.1
Other provinces				31	6
Working wards of COVID-19 patients					
Ward for mild patients				12	2.3
Ward for moderate patients				40	7.8
Ward for severe patients				162	31.5
ICU for critically ill patients				234	45.4
Ward for mixed patients				67	13
Effective working hours/per time					
<4 h				23	4.5
4–6 h				459	89.1
≧7 h				33	6.4
Expected effective working hours/per time					
≦3 h				67	13
4 h				365	70.9
5–8 h				83	16.1
Cumulative working days for COVID-19	26	19	26.3 ± 13.4		
Physical discomforts during work				375	72.8
Symptoms					
Pain				211	41
Chest distress				124	24.1
Dizziness				97	18.8
Dyspnea				236	45.8
Weakness				90	17.5
Cough				48	9.3
Faint				3	0.58
Others				55	10.7
Start time	2	2	2.4 ± 1.5		
0.5–1 h				139	27
2–4 h				317	61.6
>4 h				59	11.5
Peak time	3.5	1	3.5 ± 1.5		
0.5–1 h				49	9.5
2–4 h				340	66
>4 h				126	24.5
Interruption of work in the ward				107	20.8
Physical discomfort				61	11.8
Change the protective equipment				40	7.8
Go to the toilet				14	2.7
High mental strain				1	0.2
Others				4	0.8
End of work for COVID-19				229	44.5
According to the work management				214	41.6
Physical discomfort				8	1.6
Others				7	1.4

COVID-19, 2019 novel coronavirus; IQR, interquartile spacing; ICU, intensive care unit.

375 medical staff (72.8%) felt physical discomforts while wearing thick isolation clothes at work, mostly consist of dyspnea (236 medical staff, 45.8%), pain (211 medical staff, 41.0%), chest distress (124 medical staff, 24.1%), dizziness (97 medical staff, 18.8%), and weakness (90 medical staff, 17.5%). The onset time [mean (SD)] and peak time [mean (SD)] of these symptoms were 2.4 [1.5] hours and 3.5 [1.5] hours after working, respectively. 27.0% of the medical staff felt obvious discomforts in 1 h. 20.8% of the medical staff had been forced to leave the wards during the working time because of several reasons, including feeling physical discomforts (61 medical staff, 11.8%), changing the protective equipment (40 medical staff, 7.8%), going to the toilet (14 medical staff, 2.7%), or other reasons (5 medical staff, 1.0%). The effective working hours/per time of 459 medical staff (89.1%) was four to 6 h, and 369 medical staff (70.9%) expected the effective working hours/per time to be 4 h.

### 3.3. Feeling of the work intensity and other mental state

The mental state of the medical staff for COVID-19 was shown in Table 3. 337 medical staff (65.4%) suffered from sleep disorders, and more than half of them had 6 h or less effective sleep per day. The VAS scores [mean (SD)] of anxiety levels of the medical staff who were worried about being infected by COVID-19 were 3.8 [2.9] at work and 2.6 [2.6] during break time, respectively. Only 131 medical staff (25%) were not anxious about the COVID-19 infection during breaks, whereas 51 medical staff (10%) were highly worried about being infected by COVID-19 even during breaks. 70 medical staff (13.6%) received psychological interventions during the COVID-19 work period. The VAS score (mean [SD]) of their feeling of the work intensity levels was 6.0 [2.2] and 246 medical staff (47.8%) felt high work intensity (VAS score ≧ six). However, most of the medical staff could adapt to the COVID-19 related work with the VAS score [mean (SD)]: 2.8[2.4] (Table 3). The feeling of the work intensity were further categorized into low-moderate intensity (VAS score: zero-five) and high intensity (VAS score: six-ten). Univariate and stepwise multivariate logistic regression analyses were performed to identify potential factors that were related to the work intensity. Comparisons were made between reference category and each of the remaining groups per characteristic.

**Table 3:** Mental state of 515 COVID-19 medical staffs.

	Median	IQR	Mean ± SD	Number	%N
Sleep disorder				337	65.4
Effective daily sleep time					
≦6 h				305	59.2
7–8 h				189	36.7
>8 h				21	4.1
Anxiety levels of medical staffs from worrying about being infected by COVID-19^#^					
Work time	3	3	3.8 ± 2.9		
0 score				81	15.7
1–5 scores				333	64.7
6–10 scores				101	19.6
Break time	2	4	2.6 ± 2.6		
0 score				131	25.4
1–5 scores				333	64.7
6–10 scores				51	10
Work intensity levels of COVID-19 medical staffs^#^	5	3	6.0 ± 2.2		
0 score				11	2.1
1–5 scores				258	50.1
6–10 scores				246	47.8
Adaptability levels of COVID-19 related work^#^	2	4	2.8 ± 2.4		
0 score				124	24.1
1–5 scores				340	66
6–10 scores				51	10
Psychological intervention				70	13.6

^#^Visual Analogue Scale (VAS) was used to evaluate as in Figure 2. COVID-19, 2019 novel coronavirus; IQR, interquartile spacing.

In Table 4, the results from univariate logistic regression analysis show that none of the basic characteristics of medical staff significantly affected their feeling of the work intensity. Work location, working wards for patients with different disease severity, effective working hours/per time, effective break time, and cumulative working days were also not associated with work intensity. In contrast, sleep disorder, effective daily sleep time, and anxiety levels of being infected by COVID-19 both at work time and break time were correlated with COVID-19 work intensity (*p* < 0.05). However, after adjusting for potential confounding factors through multivariate analysis, only effective daily sleep time and anxiety levels at break time were independent related factors for work intensity (*p* < 0.05). More specifically, the medical staff who were worried about being infected by COVID-19 with a VAS score of ≥six at break time felt a significantly higher work intensity than did those with a VAS score of zero (*p* = 0.04).

**Table 4:** Related factors for work intensity of COVID-19 medical staffs^*^.

	Univariate analysis		Multivariate analysis	
	*OR* (95% CI)	*P*	*OR* (95% CI)	*P*
Age, years	1.005 (0.981–1.029)	0.70	NT	
Gender	1.064 (0.736–1.538)	0.74	NT	
Weight (kg)	1.003 (0.989–1.017)	0.66	NT	
Hometown	1.008 (0.674–1.507)	0.97	NT	
Disciplines			NT	
Intensive care unit	Reference	–		
Respiratory department	0.623 (0.348–1.118)	0.11		
Infectious disease department	1.239 (0.433–3.548)	0.69		
Emergency department	0.904 (0.267–3.058)	0.87		
Other departments	1.103 (0.754–1.615)	0.61		
Type of staff (doctors or nurses)	1.008 (0.705–1.443)	0.97	NT	
Professional titles	0.988 (0.822–1.186)	0.90	NT	
Working years	1.052 (0.831–1.330)	0.68	NT	
Physical disease	0.833 (0.432–1.609)	0.59	NT	
Work location			NT	
Hubei province	Reference	–		
Heilongjiang province	1.387 (0.969–1.985)	0.07		
Other provinces	1.065 (0.501–2.264)	0.87		
Working wards of COVID-19 patients			NT	
Ward for mild patients	Reference	–		
Ward for moderate patients	1.615 (0.375–6.951)	0.52		
Ward for severe patients	2.341 (0.611–8.966)	0.22		
ICU for critically ill patients	3.500 (0.924–13.256)	0.07		
ward for mixed patients	2.743 (0.682–11.032)	0.16		
Effective working hours/per time	1.461 (0.857–2.492)	0.16	NT	
Cumulative working days	1.007 (0.994–1.020)	0.32	NT	
Sleep disorder	1.687 (1.166–2.439)	0.00		
Effective daily sleep time	0.727 (0.535–0.987)	0.04	0.718 (0.526–0.981)	0.04
Anxiety levels of medical staffs from worrying about being infected by COVID-19^*^				
Work time	1.168 (1.097–1.244)	0.00		
0 score	Reference	–		
1–5 scores	1.552 (0.935–2.574)	0.09		
6–10 scores	3.900 (2.101–7.240)	0.00		
Break time	1.179 (1.098–1.266)	0.00		
0 score	Reference	–	Reference	–
1–5 scores	1.877 (1.234–2.856)	0.00	1.652 (0.952–2.867)	0.07
6–10 scores	4.587 (2.273–9.255)	0.00	2.503 (1.039–6.027)	0.04

^*^ Visual Analogue Scale (VAS) was used as in Figure 2, and the work intensity were categorized into low-moderate intensity (VAS score:0–5) and high intensity (VAS score:6–10); COVID-19, 2019 novel coronavirus; NT, not tested; *OR*, odds ratio; CI, confidence interval.

## 4. Discussion

As the continue increases of the confirmed COVID-19 cases worldwide, health-care systems globally could be operating at more than maximum capacity then and the health-care workers were every country’s most valuable resource (25). The medical staff were under great pressure in the early outbreak. In a district general hospital in south London, 128 (39%) of doctors experienced at least one sickness episode (26). However, there is no detailed description of the physical discomforts of the medical staff for COVID-19 during the early outbreak. Facing the unknown virus, COVID-19 medical staff kept wearing thick PPE during their work in the early stage of the outbreak. The survey showed that COVID-19 medical staff had different physical discomforts and they felt high work intensity.

The incidence of the physical discomforts related to PPE (such as dyspnea, pain, chest distress,etc.) was high in our survey and these effects were really inevitable. They are not caused by individual weakness; they are normal and expected reactions that any person will have when exposed to an unusual environment (15). Sahebi A also found that the prevalence of PPE-associated headache was relatively high, and the prevalence after and before the use of PPE was 48.27 and 30.47%, respectively (27). Adverse effects of PPE were associated with longer shift durations (28). In our study, PPE was worn for 4–6 h in 89.1% of the participants. Since the mean peak time of these physical discomforts was 3.5 h in our study, it indicates that the ideal working hours for the COVID-19 medical staff should be around 4 h every time. Also, 70.9% of them expected the effective working hours/per time to be 4 h. If PPE and human resources became sufficient, medical staff should take reasonable shifts to ensure physical health, otherwise the efficiency and quality of their work might decrease. However, due to the limitations of PPE or human resources, some of them had to work continuously for more than 6 h, which might easily cause distractions from their work. If working hours/per time cannot be shorten, some other work strategies should be applied.

Most of the medical staffs involved in the study worked for severe and critically ill patients, 162 medical staffs (31.5%) and 234 medical staffs (45.4%), respectively. When wearing thick isolate clothes, it is more difficult to perform procedures for COVID-19 patients, particularly for critically ill COVID-19 cases requiring complicated invasive procedures, such as tracheal intubation and arterial puncture/venipuncture. High frequency of performing these procedures would significantly increase the workload of the medical staffs and shorten their peak time of physical discomforts. Some measures might be beneficial for performing centralized treatments, and saving human resources, such as setting up a specialized procedure team, classifying patients being according to their severity. More work is needed to summarize and share the reasonable COVID-19 patient management.

The COVID-19 medical staff may experience considerable psychological distress due to providing direct patient care, vicarious trauma, quarantine, or self isolation (29, 30). Sleep disorders, in particular insomnia, have been commonly reported in frontline medical workers (31, 32). A meta-analysis, which included 98,533 medical staff from 71 studies, found the prevalence of insomnia among Chinese medical staff during the COVID-19 outbreak was generally high, especially for first-line workers (33). Our result showed that more than half of the medical staffs suffered from sleep disorders, and the effective daily sleep time was an independent influential factor for work intensity. These workers who had shorter effective daily sleep time during the COVID-19 work period felt higher work intensity. The medical staff were under high pressure even in the break time, which might be a major reason that lead to sleep disorder. The results indicated that only about 25% of medical staffs were not anxious about being infected by COVID-19, whereas 10% of them were highly worried about being infected by COVID-19 even during breaks. Moreover, anxiety levels of medical staffs at break time was an independent related factor for work intensity, and medical staffs with a VAS score of ≥six at break time felt a significantly higher work intensity than did those with a VAS score of zero. During the early phase of the pandemic in the Philippines, one-fourth of respondents reported moderate-to-severe anxiety and one-sixth reported moderate-to-severe depression and psychological impact (34). Besides, the workload of taking care of COVID-19 patients is very overwhelming, which challenges physical and mental limitations of medical staff all the time. Many other factors, such as change of living habit, food and environment in the isolation regions, would affect their effective sleep time, which in turn reduce the quality and productivity of their work. The risk of psychological effects from the COVID-19 pandemic is significant and manifests as stress, anxiety, depression, sleeplessness, and, in some cases, suicide (35). Therefore, it is of great importance to monitor the mental and psychological state of COVID-19 medical staffs, and provide professional psychological interventions as needed.

The psychological issues may induce healthcare workers experienced burnout during the pandemic. Ibar C found that 12% of the studied population showed burnout (52% doctors and residents, 19% nurses, 19% administrative personnel) and healthcare workers are subjected to increased levels of stress and burnout (36). Other than poor sleep, long working hours was a risk factor regarding an increase in personal burnout, work-related burnout levels and depression among health care professionals (37). The medical staff in China have been working for COVID-19 treatments in isolated areas for about 3 years. The mean continuous working days of medical staffs was 26.3 days during our survey time. However, health-care workers, unlike ventilators or wards, cannot run at 100% occupancy for long periods (25). Training workers about appropriate coping styles to adopt may be essential to enact prevention strategies to reduce burnout incidence in workers (38). Also, it is crucial to design an appropriate work schedule for medical staff, otherwise their health would be under risk and the work quality might also decrease.

Furthermore, Vancappel (39) reported that post-traumatic symptoms were also highly prevalent among French healthcare workers at the beginning of the COVID-19 crisis and they found a significant effect of the level of exposure to COVID-19 on affective symptoms. In the study by Oliver TL, the results implied that the COVID-19 pandemic had immediate effects on the eating patterns, weight changes, PA, and psychological factors of healthcare workers (40). In a large-scale survey during the COVID-19 pandemic, the results indicated that nurses who identified as women, working in ICUs, COVID-19 designated hospitals, and departments involved with treating COVID-19 patients had higher scores in mental health outcomes (41). Leaders within the hospital should investigate the working conditions and personal habits of all medical staff regularly and systematically during the COVID-19 pandemic and take any necessary preventive measures, such as improving resilience for nursing staff, in order to best care for their employees (37).

### 4.1. Study limitations

This study has several limitations. First, our investigation was carried out in the early stage of the outbreak of COVID-19 in China. The physical and mental state of the medical staff might be different in the later stage. Second, VAS score was first applied to evaluate the feeling of the work intensity of COVID-19 medical staff in this study. It was subjective and easy to implement, but further research is needed to confirm its effectiveness. Third, the details of the sleep disorders or the psychological intervention of the medical staff were not included in the questionnaire. In addition, this study fails to include the health-care workers who worked for fever clinics and who were in charge of infection surveillance. Their result of the data may be different.

## 5. Conclusion

The frontline medical staff for COVID-19 felt different physical discomforts when they wear thick isolation clothes at work in the early outbreak and they felt high work intensity. These precious data will help optimize the work management strategy to ensure the physical and mental health of medical staff in the face of similar outbreaks in future.

## Data availability statement

The raw data supporting the conclusions of this article will be made available by the authors, without undue reservation.

## Ethics statement

This study was deemed non-human-subjects research by the institutional Review Board (IRB) of the Harbin Medical University. As a result, ethical approval and written informed consent to participate in this study were not required for the study.

## Author contributions

LJ: Data curation, Formal analysis, Writing – original draft, Writing – review & editing. MY: Data curation, Writing – review & editing. HoW: Data curation, Writing – review & editing. HuW: Data curation, Formal analysis, Writing – review & editing.
