# Supplementary material for: Physical discomforts, feeling of the high work intensity and the related risk factors of the frontline medical staff during COVID-19 epidemic: an early-outbreak, national survey in China
Source: Front Public Health. 2023 Oct 12;11:1270366. doi: 10.3389/fpubh.2023.1270366 (PMC10602654; doi:10.3389/fpubh.2023.1270366)
Supplement: Supplementary file 1 [file Data_Sheet_1.docx]

**Anonymous WeChat questionnaire of front-line medical staff for COVID-19 patients**

The survey was designed to investigate the physical discomforts, the feeling of the work intensity and the related risk factors of the front-line medical staff during COVID-19 epidemic. If you agree, please complete the following questionnaire.

Are you a front-line medical staff directly taking care of the confirmed COVID-19 patients?

🞎Yes, please continue to complete the questionnaire and it will take you about 1 minute.

🞎No, you do not need to fill out this questionnaire. Thank you for your attention!

1. Your name: 🞎🞎🞎 (Only the initials of your name. For example: Zhang Xiaoming, zxm)

2. Your gender: 🞎Male 🞎Female

3. Your age: 🞎🞎🞎

4. Your weight: 🞎🞎🞎kg

5. Your hometown: _______province

6. Your discipline: 🞎ICU 🞎 Respiratory Department 🞎 Infectious Diseases Department

🞎Emergency Department 🞎Other

7. Your discipline: 🞎Doctor 🞎Nurse

8. Your professional title: 🞎Resident 🞎Attending 🞎Associate chief 🞎Chief

9. Your working years: 🞎Less than 5 years 🞎5 to 10 years 🞎More than 10 years

10. Your physical disease: 🞎None 🞎Hypertension 🞎Diabetes 🞎Other

11. Your work location: _______province

12. COVID-19 patients you worked for: 🞎Mild 🞎Moderate 🞎Severe 🞎Critical 🞎Mixed

13. Do you think the patients should be classified according to the severity? 🞎Yes 🞎No

14. Personal protective equipment (PPE) you should wear at work:

🞎medical hats:__layers 🞎N95 mask 🞎surgical mask 🞎goggles 🞎face screens 🞎a long fluid-impermeable gown 🞎an operating coat 🞎 gloves: __layers 🞎shoe covers: __layers

15. Each time you work in the COVID-19 ward :

🞎Less than 4 hours 🞎4 hours 🞎5 hours 🞎6 hours 🞎7 hours 🞎8 hours 🞎More than 8 hours

16. The expected working hours/per time in the COVID-19 ward according to your feelings:

🞎Less than 3 hours 🞎3 hours 🞎4 hours 🞎5 hours 🞎6 hours 🞎7 hours 🞎8 hours

17. Do you have any sleep disorders during the COVID-19 work period? 🞎Yes 🞎No

18. Your effective sleep time every day:

🞎Less than 6 hours 🞎6 hours 🞎7 hours 🞎8 hours 🞎More than 8 hours

19. How long have you continued working for COVID-19 patients so far? _______days

20. Do you feel any discomfort when you wear thick isolation clothes at work?

🞎No discomfort 🞎Pain (headache, facial, late hand, etc.) 🞎Chest distress 🞎Dizziness 🞎Dyspnea 🞎Weakness 🞎Cough 🞎Faint 🞎Others

21. The onset time of your discomfort from starting the work:

🞎0.5 hours 🞎1 hours 🞎2 hours 🞎3 hours 🞎4 hours 🞎5 hours 🞎6 hours 🞎7 hours

22. The peak time of your discomfort from starting the work:

🞎0.5 hours 🞎1 hours 🞎2 hours 🞎3 hours 🞎4 hours 🞎5 hours 🞎More than 5 hours

23. Have you ever been forced to leave the wards during the working time?

🞎No,never. 🞎Yes, because of feeling physical discomforts.

🞎Yes, because having to change protective equipment. 🞎Yes, because of going to the toilet.

🞎Yes, because of high mental strain. 🞎Yes, because other personal reasons.

24. Are you worried about being infected by COVID-19 during work time? Please use the VAS score (0-10): 0 no anxiety, 5 moderate anxiety and 10 severe anxiety: _______ points

25. Are you worried about being infected by COVID-19 during break time? Please use the VAS score (0-10): 0 no anxiety, 5 moderate anxiety and 10 severe anxiety: _______ points

26. How do you feel your work intensity? Please use the VAS score (0-10): 0 no anxiety, 5 moderate intensity and 10 severe intensity: _______ points

27. How do you adapt to the COVID-19 related work? Please use the VAS score (0-10): 0 easily adaptive, 5 barely adaptive and 10 hardly adaptive: _______ points

28. Have you ever been infected by COVID-19 during the COVID-19 work period? 🞎Yes 🞎No 29. Have you received any psychological intervention during the work period? 🞎Yes 🞎No

30. Have you terminated the front-line work for COVID-19 now?

🞎No, I continue my front-line work for COVID-19? 🞎Yes, because the work arrangements.

🞎Yes, because infection with COVID-19. 🞎Yes, because physical discomfort.

🞎Yes, because other reasons.

Save Submit
